# Optimization of secretion and surface localization of heterologous OVA protein in mycobacteria by using LipY as a carrier

**DOI:** 10.1186/s12934-019-1093-1

**Published:** 2019-03-06

**Authors:** Maroeska J. Burggraaf, Louis S. Ates, Alexander Speer, Kim van der Kuij, Coen Kuijl, Wilbert Bitter

**Affiliations:** 1 Medical Microbiology and Infection Control, Cancer Center Amsterdam, Amsterdam UMC, Vrije Universiteit Amsterdam, De Boelelaan 1117, Amsterdam, Netherlands; 2 Department of Experimental Immunology, Amsterdam Infection & Immunity Institute, Amsterdam UMC, University of Amsterdam, Meibergdreef 9, Amsterdam, Netherlands; 3 Molecular Microbiology, Vrije Universiteit Amsterdam, de Boelelaan 1105, Amsterdam, Netherlands

**Keywords:** Heterologous secretion, *Mycobacterium*, Vaccine, BCG, LipY, ESX, Type VII secretion

## Abstract

**Background:**

*Mycobacterium bovis* Bacille Calmette-Guérin (BCG) is not only used as a vaccine against tuberculosis but also protects against leprosy and is used as part of bladder cancer treatment to induce a protective immune response. However, protection by BCG vaccination is not optimal. To improve vaccine efficacy, recombinant BCG expressing heterologous antigens has been put forward to elicit antigen-specific cellular and humoral responses. Cell surface localized or secreted antigens induce better immune responses than their cytosolic counterparts. Optimizing secretion of heterologous proteins or protein fragments holds therefore unexplored potential for improving the efficacy of recombinant BCG vaccine candidates. Secretion of heterologous antigens requires crossing the mycobacterial inner and outer membrane. Mycobacteria have specialized ESX or type VII secretion systems that enable translocation of proteins across both membranes. Probing this secretion system could therefore be a valid approach to surface localize heterologous antigens.

**Results:**

We show that ESX-5 substrate LipY, a lipase, can be used as a carrier for heterologous secretion of an ovalbumin fragment (OVA). LipY contains a PE domain and a lipase domain, separated by a linker region. This linker domain is processed upon secretion. Fusion of the PE and linker domains of LipY to OVA enabled ESX-5-dependent secretion of the fusion construct LipY-OVA in *M. marinum*, albeit with low efficiency. Subsequent random mutagenesis of LipY-OVA and screening for increased secretion resulted in mutants with improved heterologous secretion. Detailed analysis identified two mutations in OVA that improved secretion, i.e. an L280P mutation and a protein-extending frameshift mutation. Finally, deletion of the linker domain of LipY enhanced secretion of LipY-OVA, although this mutation also reduced surface association. Further analysis in wild type LipY showed that the linker domain is required for surface association.

**Conclusion:**

We show that the ESX-5 system can be used for heterologous secretion. Furthermore, minor mutations in the substrate can enhance secretion. Especially the C-terminal region seems to be important for this. The linker domain of LipY is involved in surface association. These findings show that non-biased screening approaches aid in optimization of heterologous secretion, which can contribute to heterologous vaccine development.

**Electronic supplementary material:**

The online version of this article (10.1186/s12934-019-1093-1) contains supplementary material, which is available to authorized users.

## Background

*Mycobacterium bovis* Bacille Calmette-Guérin (BCG) is well known for its use as a vaccine against the infectious disease tuberculosis, caused by *Mycobacterium tuberculosis*. One of the major characteristics of BCG vaccination is the induction of the T-helper cell 1 (Th1)-polarized CD4^+^ T cell responses that confer cellular immune responses against bacterial infection [1, 2]. However, the efficacy of BCG vaccination is known to be suboptimal [3] and many attempts have been made to further improve vaccine efficacy. Particularly, recombinant expression of *M. tuberculosis* antigens such as 6-kDa early-secreted antigenic target (ESAT-6 or EsxA), Antigen 85B (Ag85B or FbpB) or the complete ESX-1 secretion system (of less pathogenic mycobacteria) in BCG are of great interest, as this has been shown to further enhance cellular immune responses [4–7].

Next to recombinant expression of mycobacterial proteins, expression and secretion of heterologous antigens is also of clinical interest. Expression of antigens from other pathogens in BCG has been shown to induce antigen-specific T-cell and humoral responses and to provide subsequent protection against the corresponding pathogen in vivo [8–15]. Furthermore the heterologous expression of tumor antigens has been advocated as a possible approach to improve BCG for bladder cancer treatment [16].

Heterologous expression of antigens can be accomplished in different manners. First, antigens can be expressed without making alterations to the coding region of the original gene, which usually results in cytosolic expression of the antigen. Second, antigens can be fused to a mycobacterial carrier protein that is known to be secreted by the bacteria, potentially resulting in secreted or cell surface localized antigens. Although this approach is successfully applied for Gram-negative bacteria, heterologous secretion in mycobacteria is still far from established. Carriers that have been used most frequently thus far are lipoproteins, such as the 19 kDa lipoprotein LpqH [13, 17, 18]. Next to LpqH also Ag85B is an often used carrier to secrete proteins [19, 20]. Although results were promising, surface localization and surface accessibility have not been evaluated in most studies investigating heterologous protein expression in mycobacteria, even though this is important when designing new vaccine candidates expressing heterologous antigens. The latter is illustrated by a large body of work suggesting that secreted and cell surface localized antigens evoke a better immune response than their cytosolic counterparts [13, 18, 21–23].

For successful surface localization, heterologous proteins have to cross both the inner membrane and the unique mycobacterial outer membrane. This second membrane has a similar function as the Gram-negative outer membrane, but evolved independently and contains many unique lipids such as mycolic acids. Mycobacteria use specialized secretion systems to secrete proteins across both membranes of the cell envelope. These are known as the type VII secretion systems and are named ESX-1 to ESX-5 [24]. In particular ESX-5 has been shown to secrete a large amount of proteins [25, 26]. Importantly, the type VII secretion systems secrete substrates with T-cell epitopes recognized by the host, and disruption of the ESX-5 system abolished antigen specific CD4^+^ T-cell responses against its substrates [23, 27]. Furthermore, ESX-5 is, unlike ESX-1, still present and functional in BCG, enabling its use for vaccine purposes.

Previously, we characterized the secretion, processing and surface localization of ESX-5 substrate LipY [28]. LipY is one of the very few ESX-5 substrates with a described function. This protein acts as a triacylglycerol hydrolase that utilizes triacylglycerol under hypoxic conditions and nutrient starvation [29]. LipY is a member of the PE/PPE protein family, named after the conserved proline and glutamic acid residues near their N-terminus [30]. Especially in slow growing pathogenic mycobacteria, there is a large number of PE/PPE proteins [31]. Although PE/PPE proteins lack classic secretion signals, many have been shown to be secreted in an ESX-5 dependent manner [25, 26, 32]. Furthermore, many of these proteins appear to be cell surface localized, making them an interesting carrier for heterologous secretion [33–36]. *M. tuberculosis* LipY (LipY_tub_) contains a PE-domain, followed by a linker domain and the functional lipase domain. Based on homology with the alpha/beta hydrolase fold (pfam07859) the lipase domain extends from amino acid (aa) 205 to 443. We have previously shown that the PE domain of LipY_tub_ is required for ESX-5 dependent secretion of LipY_tub_ in *M. marinum*, which was recently also confirmed by Santucci et al. [37]. Upon secretion the protein is cleaved after amino acid 149 of the linker domain. The processed C-terminal part, including the lipase domain, is then exposed at the cell surface where it carries out its function [28]. Importantly, LipY can be secreted to the cell surface in large amounts, making it a suitable carrier for heterologous secretion.

Here, we fused the PE and linker domains of LipY_tub_ to an ovalbumin fragment (OVA, a synthetic protein containing the major immunogenic epitopes of ovalbumin) to study heterologous secretion. Using mutant analysis we could show that both the LipY carrier domain and the heterologous cargo domain have an effect on secretion efficiency and membrane association. This study shows that heterologous secretion via the ESX-5 secretion system can be achieved in mycobacteria by using LipY as a carrier and provides a method to systematically improve the secretion efficiency of heterologous proteins by mycobacteria.

## Methods

### Bacterial cultures

Wild type (WT) *Mycobacterium marinum* E11 strain and ESX-5 deficient strain 7C1 with a transposon in *espG*_*5*_/*MMAR_2676* [26] were grown in 7H9 liquid medium (Difco) supplemented with 10% albumin-dextrose-catalase supplement (ADC; BD Bioscience), with or without 0.05% Tween-80, depending on the experiment, while shaking at 90 rpm at 30 °C. Solid cultures were grown on 7H10 agar plates supplemented with OADC at 30 °C. Appropriate antibiotics were used for selection (50 μg/ml hygromycin, 25 μg/ml kanamycin).

*Escherichia coli* DH5α was used for cloning experiments and was grown in LB broth or LB agar plates at 37 °C with 50 μg/ml hygromycin.

### Plasmid construction

The sequence corresponding to amino acid 1–205 from LipY was amplified from pSMT3-*lipY*_tub_ [28] using primers LipYss Fw and LipYss Rv (primer sequences are listed in Additional file 1: Table S1). OVA was amplified with a sequence coding for a C-terminal Human influenza hemagglutinin (HA)-tag from plasmid pEH3-*HbpDL*-*Glyk*-*OVAL* (personal communications, Wouter Jong) with primers OVA Fw and OVA-HA Rv. Both fragments were digested with BamHI and ligated. The ligated product was amplified with primers LipYss Fw and OVA-HA Rv and subsequently ligated into vector pSMT3 using NheI and EcoRV, resulting in pSMT3-*lipY*-*OVA*_*wt*_.

pSMT3-*LipY*_tub_ [28] was used as template for all linker deletion constructs. PCR reactions amplifying the N-terminal flanking sequences of the deletions were performed with LipY fw primer and reverse primers complementary to the respective 5′-deletion flanking sequences and a 3′-tail complementary to the 3′-deletion flanking sequences (Δ100–145 R, Δ158–205 R, Δ158–180 R, Δ181–205 R). For the C-terminal parts, a reverse primer for the HA-tag (LA RP570 (HA_R)) was combined with primers complementary to the specific truncations ((Δ100–145 F, Δ158–205 F, Δ158–180 F, Δ181–205 F). Resulting PCR products were used as template for a second PCR with primers MDTBLipY_F and LA RP570 (HA_R) to create the linker deletion LipY constructs. Products were cloned into pSMT3 by restriction with BamHI and EcoRV, creating vectors pSMT3-*lipY*Δ_100–145_, pSMT3-*lipY*_Δ158–205_, pSMT3-*lipY*_Δ158–180_ and pSMT3-*lipY*_Δ181–205_.

pSMT3_*lipY*_Δ98–201_ was constructed by overlap PCR. First, the N-terminal part flanking the deletion was amplified using primers MJB_pSMT3_65C_Fw and MJB_LD_Rv. Second, the C-terminal domain flanking the deletion was amplified with primers RU_LipY_LD_Fw and MJB_pSMT3_64C_Rv. PCR products of these PCR reactions were used as template for the overlap PCR using primers MJB_pSMT3_65C_Fw and MD_pSMT3_Fw. The resulting product was cloned into pSMT3 by restriction with BamHI and NheI.

The plasmid pSMT3-hsp60 was cloned from pSMT3-mspA Ates et al. [25]. The plasmid was digested using restriction enzymes EcoRV and NheI. The resulting backbone was isolated, blunt ended using T4DNA polymerase and re-ligated to obtain plasmid pSMT3-hsp60.

All resulting plasmids are summarized in Table 1. All vectors were transformed in *E. coli* DH5α following standard heat-shock protocol and after plasmid purification electroporated in *M. marinum* E11.

**Table 1:** Plasmids used in this study

Plasmid name	Plasmid backbone, *promotor*, selection marker	Protein product	Reference
pSMT3-*lipY*-*OVA*	pSMT3, *hsp60*, hygromycin	Rv3097c aa 1–205, OVA	This study
pSMT3-*lipY*-*OVA*_*1*_	pSMT3, *hsp60*, hygromycin	Rv3097c aa 1–205 mut 1, OVA mut 1	This study
pSMT3-*lipY*-*OVA*_*2*_	pSMT3, *hsp60*, hygromycin	Rv3097c aa 1–205 mut 2, OVA mut 2	This study
pSMT3-*lipY*-*OVA*_*3*_	pSMT3, *hsp60*, hygromycin	Rv3097c aa 1–205 mut 3, OVA mut 3	This study
pSMT3-*lipY*_*wt*_-*OVA*_*1*_	pSMT3, *hsp60*, hygromycin	Rv3097c aa 1–205, OVA mut 1	This study
pSMT3-*lipY*_*wt*_-*OVA*_*3*_	pSMT3, *hsp60*, hygromycin	Rv3097c aa 1–205, OVA mut 3	This study
pSMT3-*lipY*_*1*_-*OVA*_*wt*_	pSMT3, *hsp60*, hygromycin	Rv3097c mut 1, OVA	This study
pSMT3-*lipY*_*3*_-*OVA*_*wt*_	pSMT3, *hsp60*, hygromycin	Rv3097c mut 3, OVA	This study
pSMT3-*lipY*-*OVA*_*L280Pext*_	pSMT3, *hsp60*, hygromycin	Rv3097c aa 1–205, OVA L280P with extension caused by framehsift	This study
pSMT3-*lipY*-*OVA*_*L280P*_	pSMT3, *hsp60*, hygromycin	Rv3097c aa 1–205, OVA L280P	This study
pSMT3-*lipY*_*tub*_	pSMT3, *hsp60*, hygromycin	Rv3097c	Daleke et al. [38]
pSMT3-*lipY*-_*Δ100*–*145*_	pSMT3, *hsp60*, hygromycin	Rv3097c Δ100–145	This study
pSMT3-*lipY*-_*Δ158*–*180*_	pSMT3, *hsp60*, hygromycin	Rv3097c Δ158–180	This study
pSMT3-*lipY*-_*Δ158*–*205*_	pSMT3, *hsp60*, hygromycin	Rv3097c Δ158–205	This study
pSMT3-*lipY*-_*Δ191*–*205*_	pSMT3, *hsp60*, hygromycin	Rv3097c Δ191–205	This study
pSMT3-*lipY*-_*Δ98*–*201*_	pSMT3, *hsp60*, hygromycin	Rv3097c Δ98–201	This study

All plasmids harbor a C-terminal human influenza hemagglutinin (HA) tag. Mutations found in LipY-OVA mutant constructs can be found in Fig. 2b. *OVA* ovalbumin aa

### Error-prone PCR and screen for LipY-OVA supersecretion

To find LipY-OVA mutants that would show improved secretion, a random mutagenesis library was made. Error-prone polymerase from *Pyrococcus furiosus* [39] was used for amplification of LipY-OVA with the primers LipYss Fw and OVA-HA Rv. After 30 cycles of amplification, PCR product was isolated and cloned into pSMT3 by using NheI and EcoRV restriction enzymes. Ligation products were transformed in *E. coli* DH5α and the resulting library was plated on LB plates. About 1000 colonies were harvested and pooled from the plates and plasmids were isolated. The isolated plasmid library was electroporated into *M. marinum* E11 and cells were plated on nitrocellulose filters (0.45 μm, Merck Millipore) on 7H10 plates. Once colonies were visible, double filter assay (see below) was performed to select for colonies that showed increased secretion of LipY-OVA. Plasmids isolated from supersecreting mutants were sanger sequenced (Macrogen Europe) to detect single nucleotide polymorphisms in the insert.

### Double filter assay

To analyze secretion on double filter, bacteria were grown until cultures reached an OD_600_ of 0.5–1.0 and spotted or homogeneously distributed on nitrocellulose filter (0.45 μm, Merck Millipore) on a plate with appropriate antibiotics. Bacteria were grown at 30 °C and once colonies were visible, the filter was incubated overnight on a second filter on a fresh plate. The second filter was blocked with 5% milk in PBS and subsequently stained with a monoclonal antibody against the HA-tag (HA.11 clone 16B12, Covance) and detected via a peroxidase labelled Goat-anti-Mouse secondary antibody and 4-chloronaphthol/3,3-diaminobenzidine staining.

### Secretion analysis and immunoblot

Secretion analysis and immunoblots were performed as previously described [28]. Briefly, bacterial cultures of mid-logarithmic phase were washed with and subsequently grown overnight in 7H9 without ADC supplemented with 0.2% glycerol and 0.2% dextrose. At OD_600_ = 0.8–1.0 cells were harvested and washed with PBS. Surface-exposed proteins were extracted by 30 min incubation with 0.5% genapol X-080 (v/v; Sigma-Aldrich). For pellet samples, cells were suspended in SD buffer and disrupted by sonication. Supernatant was filtered through 0.2 μm Millipore filters and proteins were subsequently precipitated with tRNA and 10% trichloroacetic acid after overnight incubation. Protein samples were boiled and separated by SDS PAGE. After transferring proteins to nitrocellulose membranes (Merck Millipore) by Western blotting, membranes were blocked with 5% milk in PBS and incubated with antibodies raised against the HA-tag (HA 1.11), GroEL2 (CCs44, J. Belisle, NIH, Bethesda, MD, USA). Staining was performed via Goat-anti-Mouse IgG peroxidase-labelled antibodies (American Qualex Antibodies) and electro-chemi-luminescence Western Blotting Detection Reagent (Amersham Bioscience).

The immunoblots were quantified by generating optical density profiles for each lane with ImageJ. The areas under all peaks per lane were summed and normalized to the lane with the highest summed value of the blot. The colony blots were quantified by measuring the average intensity per spot with ImageJ and correct this value for the average background intensity. Spot intensity was subsequently normalized to the spot with the highest average intensity of the blot.

### Flow cytometry

Cell surface localization on whole cells was analyzed by flow cytometry. Bacteria were grown to an OD_600_ of 0.8–1.2 and were washed with 5% Bovine serum albumin (BSA) in PBS. Subsequently, bacteria were stained with antibodies raised against the HA-tag (HA.11 clone 16B12, Covance) and Goat-anti-Mouse IgG Alexa-488 (Life Technologies). After washing with 5% BSA, cells were analyzed by flow-cytometry (BD Accuri C6). Cells stained with only Goat-anti-Mouse IgG Alexa 488 were taken as control to calculate the fold change in cell surface staining.

### Lipase assay

To measure lipase activity, bacteria were grown to an OD_600_ of 0.8–1.2 in 7H9 liquid medium (Difco) supplemented with 10% ADC (BD Bioscience) and diluted to 0.5 OD_600_ in 7H9 supplemented with 10% ADC and 0.05% Tween-80. 160 μl of bacterial suspension was added to a 96-well plate together with 25 μl development solution DGGR [10 mg/ml BSA, 120 mM NaCl and 50 mM Tris pH 7.5, 1% Triton X100, 4uM 1,2-Di-O-lauryl-rac-glycero-3-(glutaric acid 6-methyl resorufin ester)]. Fluorescence was measured using a plate reader (excitation 530 nm, emission 600 nm), bottom mode. The lipase inhibitor Paraoxon (POX, 10 μM, Sigma) was used as a control for inhibition of LipY lipase activity.

### Structure prediction

To predict the structure of the lipase domain and analyze homology with other proteins aa 158–443 of LipY were analyzed by Phyre^2^ [40].

## Results

### LipY as carrier for heterologous secretion of OVA

**Fig. 1:**
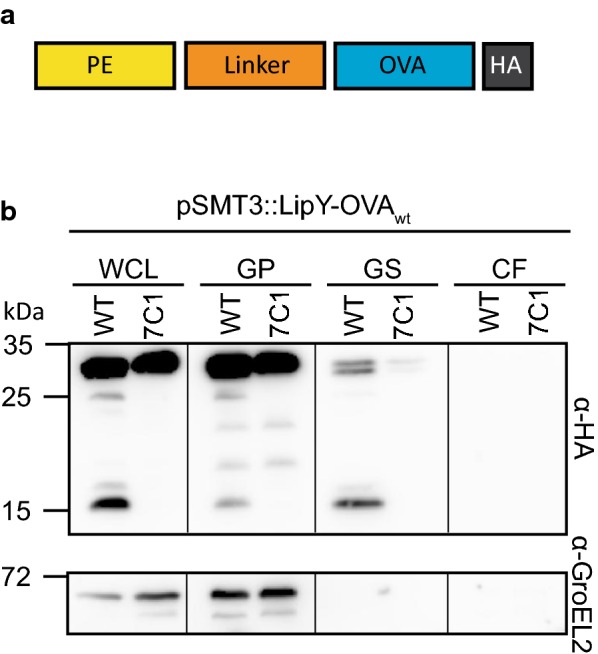
ESX-5 dependent secretion of LipY-OVA in *M. marinum*. **a** The PE domain and linker domain of LipY_tub_ are fused to OVA (amino acid 259-357 of ovalbumin) and a C-terminal HA-tag. **b** Immunoblot of secretion analysis of LipY-OVA_wt_ in *M. marinum* WT and the ESX-5 mutant *espG*_*5*_*::tn* (7C1). *WCL* Whole cell lysate, *GP* genapol pellet, *GS*, surface-enriched protein fraction genapol supernatant, *CF* culture filtrate are shown. Blot was stained with antibodies directed against HA and GroEL2 (cytosolic control)

The opportunities for heterologous secretion in mycobacteria were investigated using the ESX-5 substrate LipY as a carrier. As cargo we used an HA-tagged fragment of ovalbumin (referred to as OVA), containing amino acids 259–357 from ovalbumin, including the major known epitopes, i.e. Major histocompatibility complex (MHC) class I epitope SIINFEKL and MHC class II epitope ISQAVHAAHAEINEAGR [41, 42]. Previously, we have shown that the PE domain of LipY is required for secretion by ESX-5 and that, during or after secretion, the linker domain is cleaved after amino acid 149 [28]. Processed LipY remains associated with the cell surface. Therefore, the PE and linker domain of LipY were fused to OVA, resulting in the fusion protein LipY-OVA_wt_ (Fig. 1a).

To study surface localization of LipY-OVA_wt_, surface proteins were extracted with the mild detergent Genapol X-080. The fusion protein was readily expressed in *M. marinum*, but only a modest amount was found in the cell surface-enriched and nothing was detected in the supernatant fraction (Fig. 1b). Analysis of LipY-OVA_wt_ in ESX-5 transposon mutant 7C1 (*espG*_*5*_*::tn*) showed that this modest secretion of LipY-OVA_wt_ was ESX-5 dependent, resembling the secretion of LipY_tub_. Thus, LipY can serve as a carrier for heterologous secretion of OVA in an ESX-5 dependent manner, although the efficiency is rather low.

### Mutations in OVA can enhance secretion of LipY-OVA in *M. marinum*

Secretion, or surface localization, of mycobacterial antigens is required for the induction of specific CD4^+^ T-cell responses [27, 43, 44] and is therefore a crucial factor to optimize vaccine design. To investigate whether we could enhance secretion of LipY-OVA_wt_, we introduced random point mutations in the gene coding for LipY-OVA_wt_ by amplification of the DNA fragment with error-prone DNA polymerase. Subsequently, a library of these error-prone constructs were introduced in *M. marinum* and colonies were screened for increased secretion of LipY-OVA by double filter screening.

**Fig. 2:**
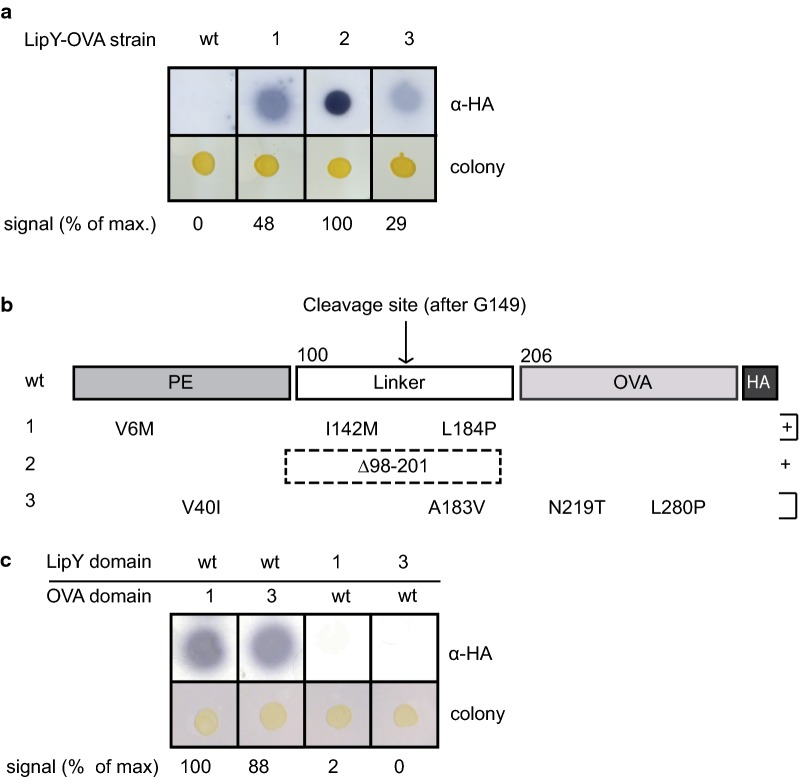
Error-prone PCR mutants of LipY-OVA show increased secretion in *M. marinum*. **a** Secretion of LipY-OVA_wt_, LipY-OVA_1_, LipY-OVA_2_ and LipY-OVA_3_ was analyzed by double filter assay. Proteins were visualized by anti-HA. **b** Sequencing of constructs showed different mutations in both LipY and OVA domains. LipY-OVA_1_ and LipY-OVA_3_ show an extension of the HA-tag due to frameshift mutations. **c** Double filter assay showing secretion of LipY-OVA swap constructs by *M. marinum*. Mutant LipY and OVA domains were swapped with wild type domains. Total amount of HA-labeled protein was calculated using ImageJ Gel Analysis tools and the highest score was set to 100%

We identified three LipY-OVA clones with increased secretion (Fig. 2a). Sequencing of the mutated constructs isolated from these clones revealed multiple mutations (Fig. 2b). Whereas LipY-OVA_wt_ was not secreted on double filter, mutants 1 (LipY-OVA_1_), 2 (LipY-OVA_2_) and 3 (LipY-OVA_3_) showed secretion. Especially mutant 2 (LipY-OVA_2_) showed a strong increase in secretion, e.g. twofold higher than LipY-OVA_1_. Strikingly, both LipY-OVA_1_ and LipY-OVA_3_ had independent frame shift mutations in the same codon, resulting in a nineteen amino acid extension after the HA-tag (Table 2). In LipY-OVA_1_ the extension was caused by a 2 basepair (bp) frameshift deletion in the last two codons before the stop codon. In LipY-OVA_3_ a 1 bp insertion in the penultimate codon caused a frameshift resulting in a nearly identical extension of the HA-tag. The most striking alteration was located in LipY-OVA_2_, instead of a single basepair change we observed a deletion that removed aa 98–201 of the gene, which spans almost the complete LipY linker domain, leaving only the PE domain in front of OVA.

**Table 2:** Independent polymorphisms lead to a C-terminal extension of LipY-OVA

Construct	C-terminal amino acid sequence	Deletion/insertion (bp)
LipY-OVA_wt_	YPYDVPDYA	–
LipY-OVA_1_	YPYDVPDY*LRYLERPPPRCEPDLVIMS*	Δ942T and Δ943G
LipY-OVA_3_	YPYDVPDYA*LRYLERPPPRCEPDLVIMS*	+ 944C

Depicted is the C-terminal amino acid sequence of LipY-OVA_wt_ containing the HA-tag. Constructs LipY-OVA_1_ and LipY-OVA_3_ had independent deletions/insertions (column 3) leading to a similar C-terminal extension after the HA-tag (Column 2, shown in italics). Proline triplet is underlined

To determine which mutation was responsible for the increased secretion we first exchanged either the LipY-encoding domain for the original unmutated version (LipY_WT_) or the original OVA-encoding domain (OVA_WT_). Whereas LipY_wt_-OVA_1_ and LipY_wt_-OVA_3_-showed high secretion levels, similar to LipY-OVA_1_ and LipY-OVA_3_, secretion of LipY_1_-OVA_wt_ and LipY_3_-OVA_wt_ was not detectable (Fig. 2c). Therefore, we concluded that the mutations in the OVA domain are responsible for the enhanced secretion phenotype. Importantly, expression of the fusion constructs was similar between different mutants (Additional file 2: Figure S1). Notably, LipY_wt_-OVA_1_ exhibited a single mutation, i.e. the extension of the OVA domain, indicating that this change is sufficient to enhance the signal on double filter. This is not entirely surprising, as we identified two independent mutations that resulted in exactly the same frameshift.

A notable feature of the extension in LipY-OVA_1_ and LipY-OVA_3_ was the presence of three consecutive prolines (underlined in Table 2). Such a proline repeat can cause stalling of the ribosome [45], possibly leading to better folding and enhanced secretion of LipY-OVA. To test this hypothesis we replaced the proline triplet by three alanines, resulting in LipY-OVA_1(PPP/AAA)_. Double filter analysis showed however that LipY-OVA_1(PPP/AAA)_ was secreted to a similar extent as LipY-OVA_1_, indicating that enhanced secretion is not caused by the proline triplet (Additional file 3: Figure S2).

### Mutation L280P enhances secretion and surface localization of LipY-OVA

**Fig. 3:**
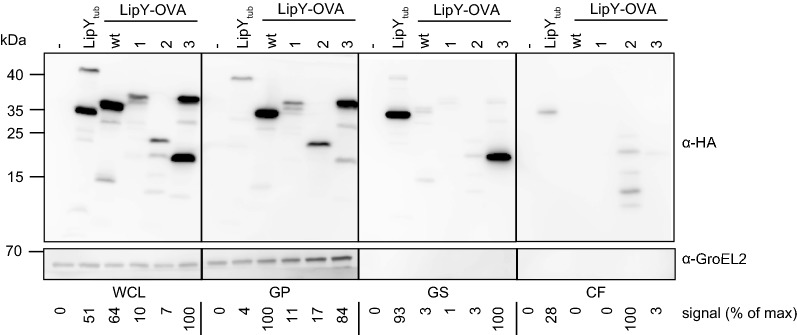
Secretion analysis of LipY-OVA mutants. Immunoblots of whole cell lysate (WCL), genapol pellet (GP), genapol supernatant (GS) and culture filtrate (CF) of *M. marinum* WT, LipY_tub-_, LipY-OVA_wt_, LipY-OVA_1_, LipY-OVA_2_ and LipY-OVA_3_. Proteins were visualized with anti-HA and anti-GroEL2 (cytosolic control). Total amount of HA-labeled protein was calculated for each blot separately using ImageJ Gel Analysis tools and the highest score was set to 100%, taking into account all bands in the lane

To analyze the localization of LipY-OVA in more detail, we performed secretion analysis of the different mutants with improved secretion on double filter using liquid cultures. Secreted mycobacterial proteins can be found either in the supernatant or in the cell surface-enriched fraction, which is obtained after washing harvested cells with the mild detergent Genapol X-080. All chimeric proteins were expressed, levels ranging from 0.1 to 2 fold as compared to LipY_tub_ (Fig. 3). LipY-OVA_2_ was the only fusion protein that could be detected in modestly increased amounts in the culture supernatant. Although the total amount of LipY-OVA_2_ found in the supernatant was approximately fourfold higher than LipY_tub_, this modified fusion protein showed various degradation products. This indicates that our mutants are poorly released from the mycobacterial surface into the culture medium, or that they are not stable in culture medium. Evidence for the latter hypothesis is the presence of degradation products for LipY-OVA_2_ in the supernatant fraction. Instability of released proteins is also observed for EspE [46].

The picture was different for the cell surface-enriched fraction. In this fraction the amount of LipY-OVA_3_ was 30 fold higher than LipY-OVA_wt_ (Fig. 3). Interestingly, it was also present in higher amounts than LipY-OVA_1_, although both mutants showed similar secretion phenotypes on double filter. In addition, LipY-OVA_3_ showed a band with higher mobility in the surface-enriched fraction, indicating processing during or after the translocation to the cell surface. This processed band resembles the processing seen for full-length LipY, corresponding with cell surface localization of LipY. Hence, these results indicate that LipY-OVA_3_ is the most efficiently translocated protein.

**Fig. 4:**
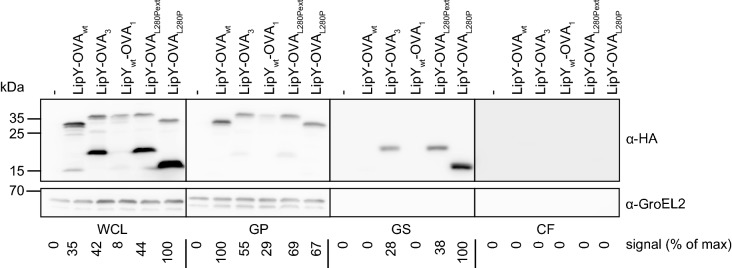
Secretion analysis of LipY-OVA L280P mutation. Immunoblot of whole cell lysate (WCL), genapol pellet (GP), genapol supernatant (GS) and culture filtrate (CF) of *M. marinum* WT, LipY-OVA_wt_, LipY-OVA_3_, LipY_wt_-OVA_1_, LipY-OVA_L280Pext_ and LipY-OVA_L280P_. Proteins were visualized by anti-HA-tag and anti-GroEL2 (cytosolic control). Total amount of HA-labeled protein was calculated for each blot separately using ImageJ Gel Analysis tools and the highest score was set to 100%, taking into account all bands in the lane

Next, we aimed to determine which mutation(s) in LipY-OVA_3_ were responsible for the increased secretion and surface localization. Secretion analysis of the previously constructed pSMT3-*lipY*_*wt*_-*OVA*_*3*_ construct showed that the mutations in the OVA_3_ domain were responsible for the presence of LipY-OVA_3_ on the cell surface (Fig. 4). To further elucidate the role of the individual mutations, i.e. N219T, L280P and the extension of the HA-tag present in the OVA-domain of LipY-OVA_3_, we created LipY-OVA constructs with these polymorphisms. Western blot analysis of LipY-OVA_L280P_ and the same construct with the C-terminal extension (LipY-OVA_L280Pext_) showed secretion and subsequent surface localization of the processed form, resembling the phenotype found for LipY-OVA_3_ (Fig. 4, genapol supernatant). Secretion of LipY-OVA_L280P_ and LipY-OVA_L280Pext_ was even a bit more efficient than LipY-OVA_3_ (respectively 1.3 and 3 fold higher). In contrast, LipY-OVA_wt_ was mainly present in its full length form and was only partially processed (15 kDa band, 7% of total expression) (Fig. 4, whole cell lysate). In conclusion, mutation L280P appeared to be sufficient to markedly enhance secretion and surface localization of LipY-OVA, indicating that modest cargo modification can be sufficient for improving secretion efficiency.

Since application of heterologous secretion for vaccine strategies would ultimately require expression in BCG, the selected LipY-OVA mutant constructs were also introduced in BCG. All these LipY-OVA constructs were expressed at comparable levels (Additional file 4: Figure S3) in BCG. LipY-OVA could be observed in the surface-enriched fraction (GS), but only after relatively long exposure times. Similar to the observations in *M. marinum*, the amount of LipY-OVA_3_ was higher than LipY-OVA_wt_ (twofold), whereas the amount in the pellet fraction was less for this mutant (fivefold). The other two chimeras, LipY-OVA_1_ and LipY-OVA_2_ were not increased in the surface-enriched fraction as compared to LipY-OVA_wt_. As published before [28], LipY_tub_ was not secreted by BCG in vitro, as was shown by the absence of the processed band (35 kDa) in all fractions.

### Surface exposure of LipY-OVA on intact cells

**Fig. 5:**
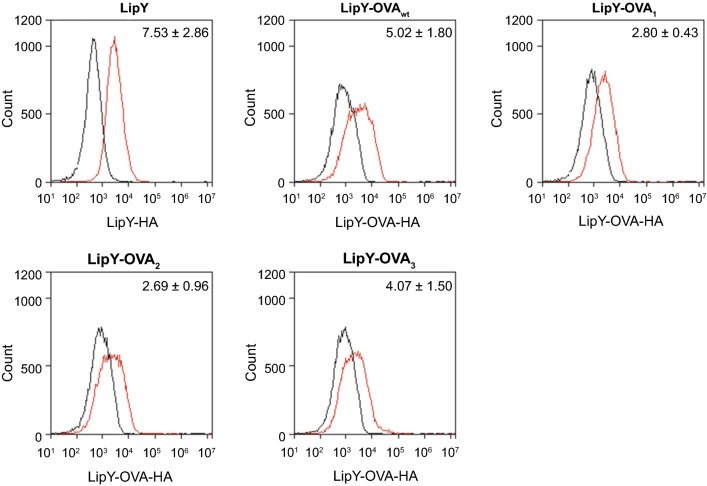
Flow cytometry results of surface labeling of LipY and LipY-OVA. *M. marinum* cells were labeled for LipY-HA or LipY-OVA-HA and measured by flow cytometry. Fold change averages of the median fluorescence from wild-type (black line) to LipY-HA or LipY-OVA-HA (red line) and standard deviation over 3 independent experiments are depicted. Plots show results of one representative experiment

Surface localization does not necessarily equal surface accessibility, since proteins that are detected in denatured conditions in cell surface fractions might be shielded by other capsular components and are therefore not exposed to the exterior of the cell. Therefore, we analyzed surface accessibility of LipY-OVA_1_, LipY-OVA_2_ and LipY-OVA_3_ in *M. marinum* on intact cells by immunolabeling and flow cytometry. All LipY-OVA constructs showed surface labeling, as did full-length LipY_tub_ (Fig. 5). The increase in surface signal suggested that LipY_tub_ (7.5 fold increase) and LipY-OVA_3_ (4.1 fold increase) have a higher surface labeling as compared to LipY-OVA_1_ and LipY-OVA_2_ (2.8 and 2.7 fold increase, respectively). This resembles the subcellular fractionation data, in which LipY_tub_ and LipY-OVA_3_ were present in significant amounts in the genapol supernatant fraction. Unexpectedly, no clear difference was seen between the surface labeling of LipY-OVA_wt_ (5.0 fold increase) and LipY-OVA_3_, despite the clearly enhanced presence of LipY-OVA_3_ in the cell surface-enriched fraction. This might be explained by the observation that LipY-OVA_3_ is mainly present in its processed form. Due to the orientation and/or length of the processed protein, the HA-tag could be less well accessible on the cell surface for antibody labeling.

### Deletion of the linker domain reduces cell surface localization of LipY

**Fig. 6:**
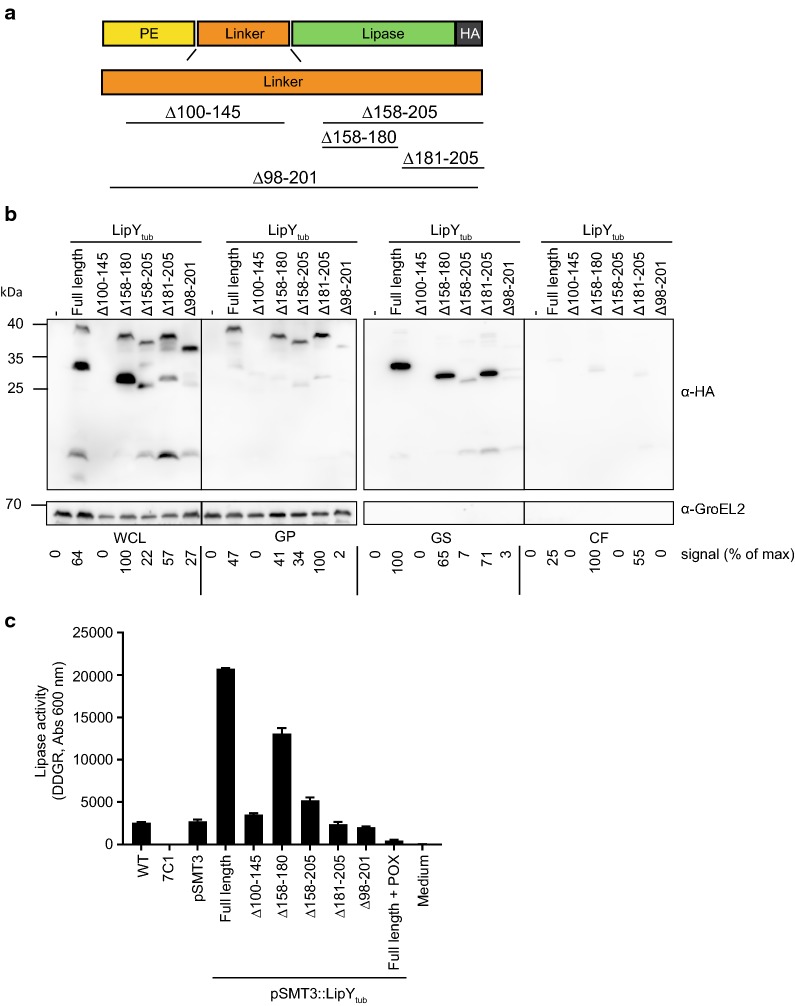
The effect of linker domain deletions in LipY_tub_. **a** Schematic representation of deletions that were made in the linker domain of LipY. **b** Immunoblot showing whole cell lysate (WCL), genapol pellet (GP), genapol supernatant (GS) and culture filtrate (CF) of *M. marinum* expressing LipY_tub_ and LipY_tub_ linker domain deletion mutants. Proteins were visualized with anti-HA and anti-GroEL (cytosolic control). Total amount of HA-labeled protein was calculated for each blot separately using ImageJ Gel Analysis tools and the highest score was set to 100%, taking into account all bands in the lane. **c** Lipase activity was measured on whole cells *M. marinum* by using DGGR as a substrate. One representative experiment is shown out of three independent experiments that showed similar results

LipY-OVA_2_ showed a deletion of aa 98–201, which is almost completely coinciding with the predicted linker domain (aa 100–205). This mutant showed a higher level of secretion, as was shown by both double filter assay (Fig. 2a) and supernatant precipitation (Fig. 3). These results suggest a role for the linker domain in surface localization. To further investigate this, different parts of the linker domain (aa 100–205) were deleted in the original LipY_tub_ protein and analyzed in *M. marinum* (Fig. 6a). To retain the known cleavage site (after aa 149) in the partial linker deletions, we constructed LipY_Δ100–145_ and LipY_Δ158–205_. In addition, two smaller deletions of the C-terminal part of the linker domain were constructed based on the conservation of amino acids between *M. marinum* and *M. tuberculosis* LipY, i.e. either the less conserved amino acids 158–180 were deleted in LipY_Δ158–180_, or the highly conserved amino acids 181–205 were deleted to obtain LipY_Δ181–205_. Finally, the deletion found in LipY-OVA_2_ (aa 98–201) was mimicked in LipY_tub_ (LipY_Δ98–201_). Expression and secretion of all LipY deletion constructs were analyzed in *M. marinum*. Deletion of the N-terminal part of the linker domain (LipY_Δ100–145_) did not result in a stable product and was not observed on double filter nor upon cell fractionation. This finding resembles previous data, which showed that the PE-domain with only the first 25 aa of the linker domain alone is not stable [28]. All other linker domain mutants were reasonably well expressed and were further analyzed by cell fractionation (Fig. 6b). These mutants showed expression of the full length protein in the whole-cell lysate. LipY_Δ158–180_, LipY_Δ158–205_ and LipY_Δ181–205_ also showed smaller bands that correspond to their relative molecular weight after processing after amino acid 149. Surprisingly, processing was also observed for LipY_Δ98–201_, although this construct lacks the protease cleavage site at amino acid 149 in the linker. This suggests that the PE-domain is still removed, since the length of the smaller band corresponded to the predicted molecular weight of the lipase domain only, which is in line with previous observations [28].

The processed bands of LipY_Δ158–180_ and LipY_Δ181–205_ were mainly found in the genapol X-080 supernatant, indicating that they were processed and retained at the cell surface. In comparison, LipY_Δ158–205_ was present in much lower amounts in this fraction. Apparently, partial deletion of the C-terminal part of the linker domain (aa 158–180 or aa 181–205) did not affect cell surface localization, while deletion of the whole C-terminal part (aa 158–205) reduced surface localization.

Together these results indicate that in *M. marinum* the C-terminal part of the linker domain is involved in retention of LipY to the surface. Deletion of the full linker domain reduced surface localization of both LipY_Δ98–201_ and LipY-OVA_2_. However, in contrast to LipY-OVA_2_, culture supernatant levels of LipY_Δ98–201_ did not increase.

### The linker domain is involved in lipase activity of LipY

To examine whether the linker domain is also important in LipY activity, we assessed the lipase activity of the different linker deletion mutants. Lipase activity was measured using a whole cells assay. Lipase activity was almost reduced to zero in a secretion deficient ESX-5 negative background (Fig. 6c). Introduction of a plasmid containing the *lipY* gene results in significantly increased lipase activity, which can be reduced again by addition of the lipase inhibitor POX. The lack of activity of LipY_Δ100–145_ is likely due to the instability of the construct. Surprisingly, also most other deletion mutants did not show any lipase activity exceeding the background lipase activity of *M. marinum* WT even though production levels of these variants were in fairly similar amounts. Only LipY_Δ158–180_ still showed lipase activity. This was most clear for LipY_Δ181–205_, which seems in amounts comparable to intact LipY, but which showed no lipase activity above background levels. This means that, even though partial deletions of the linker domain do not affect surface localization of LipY, lipase activity is severely hampered, especially when the C-terminal part of the linker domain is removed.

## Discussion

The BCG strain is one of the most widely used vaccines worldwide. Because of the immunogenic properties of BCG that induce clear cellular immunity against many intracellular pathogens, several attempts have been made to enhance vaccine efficacy by recombinant expression of mycobacterial and non-mycobacterial antigens [5]. Since secretion of the antigens increases the adaptive immune response against *Mycobacterium tuberculosis* antigens [43], we aimed to optimize heterologous secretion in mycobacteria by using the ESX-5 system and assessing both secretion and cell surface localization of a non-mycobacterial protein fragment of OVA.

By using LipY as a carrier we provided a proof of principle that the ESX-5 system could indeed be used for heterologous secretion in *M. marinum,* although secretion efficiencies were reduced as compared to the wild-type protein. By introducing random mutations in LipY-OVA we were able to identify mutants that showed enhanced secretion and surface localization, indicating that this is a viable approach that could be used for other heterologous constructs. These experiments showed two different mutations in the cargo that improved secretion. The first group of mutations had their stop codon removed by small insertions or deletions. Remarkably, both these alterations gave rise to the same frameshift resulting in an extension containing a proline triplet. Proline triplets can in theory be responsible for enhanced secretion, by securing better folding of the protein due to ribosome stalling [45]. However, further analysis showed that the proline triplet was not responsible for the increased signal. At this point, we cannot exclude that extension of the HA-tag increased detection by the antibody, for example by affecting protein folding.

The second mutation improving secretion was L280P in the OVA-domain. In addition to increased secretion, we found that LipY-OVA_L280P_ induced processing of LipY-OVA in a way similar to the processing seen for LipY. Particularly, this processed form showed increased surface localization. Most likely, this increased surface localization and subsequent processing are the result of enhanced secretion through the ESX-5 system. Prolines are known to induce kinks in peptides [47], which could make the OVA-domain more suitable for secretion through the ESX-5 system. This experiment shows that a single mutation in the OVA-domain can be sufficient for enhanced secretion by the ESX-5 system. This confirms the expectation that there are restrictions on protein size and/or conformation of ESX-5 substrates [48]. On the other hand, our experiments also show that already minor mutations can enable secretion of heterologous proteins through the ESX-5 system. Obviously, these mutations will be specific for each potential substrate and might change substrate structure, but do not necessarily have to change substrate function or immunogenic epitopes. This is illustrated by mutation L280P, which increased processing but is not part of the MHC-I or MHC-II epitopes present in the OVA domain.

Surface localization of LipY-OVA was further analyzed by immunolabeling of whole cells. Whereas surface localization was well visible after secretion analysis on western blot, the differences on whole cells, analyzed with flow cytometry, were relatively small. This might be due to reduced accessibility of the HA-tag in a folded state on the cell surface and putative shielding by the mycobacterial capsule. This could explain why LipY-OVA_3_ was detected in high amounts in the genapol-extracted fraction in denatured form, but not on the surface of intact cells. Nevertheless, LipY-OVA was clearly present on the cell surface, although cell surface accessibility did not significantly differ between mutants.

The absence of LipY secretion by BCG in culture most likely resembles previous observations in *M. tuberculosis* [28]. This bacterium only secretes LipY and shows lipase activity upon nutrient starvation or hypoxia, which might also explain the low levels of LipY-OVA in the surface-enriched fraction. Possibly one or more yet unknown proteins required for efficient processing, secretion and surface localization of LipY are not expressed in BCG in nutrient rich in vitro conditions.

The deletion of the linker domain (aa 98–201) enhanced secretion of LipY-OVA. This compelled us to study the functional role of the LipY linker domain in *M. marinum*. It is likely that surface localization is linked to processing of the linker domain, since LipY-OVA was retained at the cell surface only after processing of the linker domain, as seen in LipY-OVA_L280P_. These data correspond well with our previously published model of LipY_tub_ secretion, in which the protein is cleaved after Gly-149, after which the C-terminal part stays associated with the cell surface [28]. Further analysis of the linker domain of LipY_tub_ showed that when the major part of the linker domain (aa 98–201) or its C-terminal part (aa 158–205) was deleted, surface localization was severely reduced.

Although surface localization and processing of LipY_Δ158–180_ and LipY_Δ181–205_ were only slightly affected, especially LipY_Δ181–205_ showed strongly reduced lipase activity in whole cells. Previously, it has been shown that the PE domain negatively regulates lipase activity [37, 49, 50]. Our data suggests that on whole cells also the C-terminal part of the linker domain is (in)directly involved in lipase activity. One option is that this domain is important for the folding of the actual lipase domain. More detailed analysis of the lipase domain (Phyre^2^ [40]) indicated that the lipase domain itself could be extended to aa 192, which would include an extra loop that might be important for proper functioning of the lipase domain. Another option is that the C-terminal aa of the linker domain serve as a platform or stem-like structure that is necessary to make the active site of the lipase domain accessible to the substrate at the cell surface. Since the mycobacterial cell wall is enveloped by a capsular layer of proteins and polysaccharides certain distance might be needed to bring the lipase domain in contact with the extracellular environment [51].

While further research is needed to optimize LipY-mediated heterologous secretion in BCG, this study shows that ESX-5 substrate LipY can act as a carrier for secretion of heterologous proteins both in *M. marinum* and BCG. This paves the way to improve recombinant expression approaches that already have been taken to improve vaccine efficacy. More importantly, we show a viable and affordable method for optimization of heterologous secretion in mycobacteria by combining error-prone PCR with double filter screening methods. In principle, this method can be adopted to any carrier-cargo sequence to find individual mutations required for enhanced secretion, which in turn may lead to improved performance of recombinant mycobacterial vaccine candidates. If one could combine this screen with Flow Cytometry Cell Sorting many more mutants could be analyzed, although one would miss mutants with improved secretion without increased surface exposure, such as LipY-OVA_2_.

## Conclusions

Here we have shown that the mycobacterial ESX-5 system can serve as a route for heterologous secretion. By fusing the PE domain and linker domain of ESX-5 substrate LipY to a fragment of OVA, OVA was secreted in an ESX-5 dependent manner in *M. marinum*. Subsequent random mutagenesis of the fusion protein LipY-OVA showed that minor changes in the heterologous substrate can enhance secretion. In addition deletion of the linker domain of LipY-OVA enhanced secretion, but reduced surface association. In agreement with the latter finding, the linker domain was also shown to be important for surface localization of intact LipY. Together, this work is the first to methodologically analyze and optimize heterologous secretion in mycobacteria. This could give new momentum to recombinant BCG development, with implications for recombinant vaccines and BCG bladder cancer treatment.

## Additional files

**Additional file 1: Table S1.** Primers used in this study.

**Additional file 2: Figure S1.** Expression of LipY-OVA swap constructs in *M. marinum.* LipY-OVA swap mutants showed similar expression levels, as was analyzed by collecting pellet fractions and staining with anti-HA and anti-GroEL2 (cytosolic control).

**Additional file 3: Figure S2.** Double filter assay of LipY-OVA_PPP/AAA_ Double filter assay showing LipY-OVA in which the proline triplet in the extension of LipY-OVA_1_ was replaced by an alanine triplet. HA-tagged proteins were detected by immunoblotting with anti HA.

**Additional file 4: Figure S3.** Secretion analysis of LipY-OVA in *M. bovis* BCG. LipY_tub_ and LipY-OVA constructs were expressed in BCG. Whole cell lysate (WCL), genapol pellet (GP), genapol supernatant (GS) and culture filtrate (CF) were collected and analyzed on western blot. Proteins were visualized by anti-HA-tag and anti-GroEL2 (cytosolic control). Proteins in genapol supernatant were only visible after long exposure (exposure time pellet fraction = 0.3 s, surface-enriched fraction = 10 s). Total amount of HA-labeled protein was calculated for each blot separately.

## Abbreviations

aa: amino acid
BCG: Bacille Calmette-Guérin
bp: basepair
*E. coli*: *Escherichia coli*
HA: human influenza hemagglutinin
*M. marinum*: *Mycobacterium marinum*
*M. tuberculosis*: *Mycobacterium tuberculosis*
OVA: amino acid 259–357 of ovalbumin
Th1: T-helper cell 1
WT: wild type
