# Supplementary material for: Optimization of secretion and surface localization of heterologous OVA protein in mycobacteria by using LipY as a carrier
Source: Microb Cell Fact. 2019 Mar 6;18:44. doi: 10.1186/s12934-019-1093-1 (PMC6402100; doi:10.1186/s12934-019-1093-1)
Supplement: Supplementary file 1 — Additional file 1: Table S1. Primers used in this study. [file 12934_2019_1093_MOESM1_ESM.docx]

**Table 1 Primers used in this study**

| **Primer name** | **Primer sequence 5’ 🡪 3’** |
| --- | --- |
| LipYss Fw | TTTGCTAGCGTGGTGTCTTATGTTGTTGC |
| LipYss Rv | AAAGGATCCGTTCAGCAACGGCGGTGGGG |
| OVA Fw | TTTGGATCCGTTAGCGGTCTGGAACAGCT |
| OVA-HA Rv | AAAGATATCTTAAGCATAATCAGGAACATCATACGGATATGCTTCTGCGCTACCAACAA |
| Δ100-145 F | TGGCCAACGCTTCGCTGTTGCGCAGGCAGGGATCTTCACC |
| Δ100-145 R | GGTGAAGATCCCTGCCTGCGCAACAGCGAAGCGTTGGCCA |
| Δ158-205 F | CCGGATTCGACATCGCTTCGTCGCTGCTGGGACAGACGGT |
| Δ158-205 R | ACCGTCTGTCCCAGCAGCGACGAAGCGATGTCGAATCCGG |
| Δ158-180 F | CCGGATTCGACATCGCTTCGCTTTTAGCGCTCATTGCCAG |
| Δ158-180 R | CTGGCAATGAGCGCTAAAAGCGAAGCGATGTCGAATCCGG |
| Δ181-205 F | ACTTTGCAATCCCAAACAATTCGCTGCTGGGACAGACGGT |
| Δ181-205 R | ACCGTCTGTCCCAGCAGCGAATTGTTTGGGATTGCAAAGT |
| LA RP570 (HA_R) | ATGGATCCTTAAGCATAATCAGGAACATCATA |
| MDTBLipY_F | ACTGCTAGCGTGGTGTCTTATGTTGTT |
| MJB_pSMT3_65C_Fw | CCCGGCCAGCGTAAGTAGCGG |
| MJB_LD_Rv | TCAGCAACGGCGAAGCGTTGGCCAGCTC |
| RU_LipY_LD_Fw | AGCTGGCCAACGCTTCGCCGTTGCTGAACTCGCTGCTGGGACA |
| MJB_pSMT3_64C_Rv | GATTACCAGATCTGGCTCGAGCTCCACC |
| MD_pSMT3_Fw | AGTGAGCGCAACGCAATTA |
