# Supplementary figures and images for: Optimization of secretion and surface localization of heterologous OVA protein in mycobacteria by using LipY as a carrier
Source: Microb Cell Fact. 2019 Mar 6;18:44. doi: 10.1186/s12934-019-1093-1 (PMC6402100; doi:10.1186/s12934-019-1093-1)

LipY domain

wt

wt

1

3

OVA domain

1

3

wt

wt

-

kDa

35

25

70

$\alpha$ -HA

$\alpha$ -GroEL2

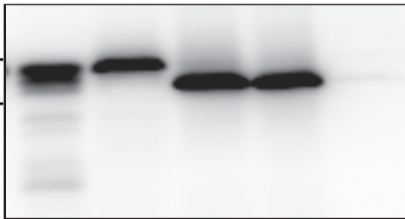

Supplement: Supplementary file 2 — Additional file 2: Figure S1. Expression of LipY-OVA swap constructs in M. marinum. LipY-OVA swap mutants showed similar expression levels, as was analyzed by collecting pellet fractions and staining with anti-HA and anti-GroEL2 (cytosolic control). [file 12934_2019_1093_MOESM2_ESM.pdf]
